# Study on soil-water characteristic curves in the profiles of collapsing walls of typical granite Benggang in southeast China

**DOI:** 10.7717/peerj.13526

**Published:** 2022-06-01

**Authors:** Liting Zhang, Shujun Sun, Mengqi Lin, Kaijun Feng, Yue Zhang, Jinshi Lin, Hongli Ge, Yanhe Huang, Fangshi Jiang

**Affiliations:** College of Resources and Environment, Fujian Agriculture and Forestry University, Fuzhou, Fujian, China

**Keywords:** Gravity erosion, Soil physics, Soil water potential, Undisturbed soil, Weathering crust

## Abstract

Benggang with steep collapsing walls is one of the worst soil erosion problems in South China. The collapse of walls is the most critical process in Benggang development. This is mainly due to the soil water properties. The soil water characteristic curve (SWCC) is a key indicator for analyzing soil moisture, but the SWCC and its mechanism of influence in collapsing walls remain obscure. A pressure plate meter was used for drying experiments to research the SWCCs of undisturbed soils of five layers (from top to bottom: red soil layer, transition layer I, sand soil layer, transition layer II and detrital layer) of two typical collapsing walls. The van Genuchten (VG) model can be fitted to the SWCCs for different layers (NSE ≥ 0.90). With increasing soil depth, the parameters *a* and *θ*_s_ first decreased and then increased, the parameters *n* first increased and then decreased, *θ*_r_ declined as the soil depth increased. These findings illustrate that soil water holding capacity decreases with increasing soil depth. The bottom of the soil is weak in water retention and water can easily reach saturation, resulting in a decline in soil stability, thus promoting soil collapse and finally inducing upper soil collapse. Furthermore, gravel content and particle morphology are factors that should not be neglected for SWCCs. The results of this study provide a theoretical basis for understanding the process of wall collapse in Benggang landforms in South China.

## Introduction

Bangang is a typical gully erosion type in China, with steep wall collapse (steeper than 65°); it is also called collapsing gullies, and it is widely distributed in tropical and subtropical granite areas of South China (Xu, 1996; Wen et al., 2020a). Survey data show that the total number of Benggang in the seven provinces of Jiangxi, Guangdong and other regions in southeastern China exceed 239,100, and ninety percent of them are highly active and relatively difficult to control (Zhong et al., 2013; Huang et al., 2019). The overall area is also relatively large, over 1,200 km^2^ in area (Liao et al., 2020). These gullies develop very quickly and often erupt in a short period of time; the average erosion modulus exceeds 500 Mg ha^−1^ y^−1^. Erosion of these gullies is much faster than that of other slopes, more than 50-fold faster than that of flat slopes and slopes with high vegetation coverage (Zhong et al., 2013). The rapid development of Benggang is associated with a high risk of sudden-onset disasters and large soil losses. Benggang often suffers from natural disasters such as floods and mud-rock flows, which seriously affect the sustainable development of local areas, causing irreversible harm to some areas with gullies. From 1950 to 2005, Benggang resulted in the loss of 60 MT of soil due to natural disasters. Soil erosion has caused serious damage to farmland, houses, water tanks and ponds, with cumulative economic losses exceeding 3.28 billion USD and over 9.17 million people affected (Jiang et al., 2014).

**Figure 1:**
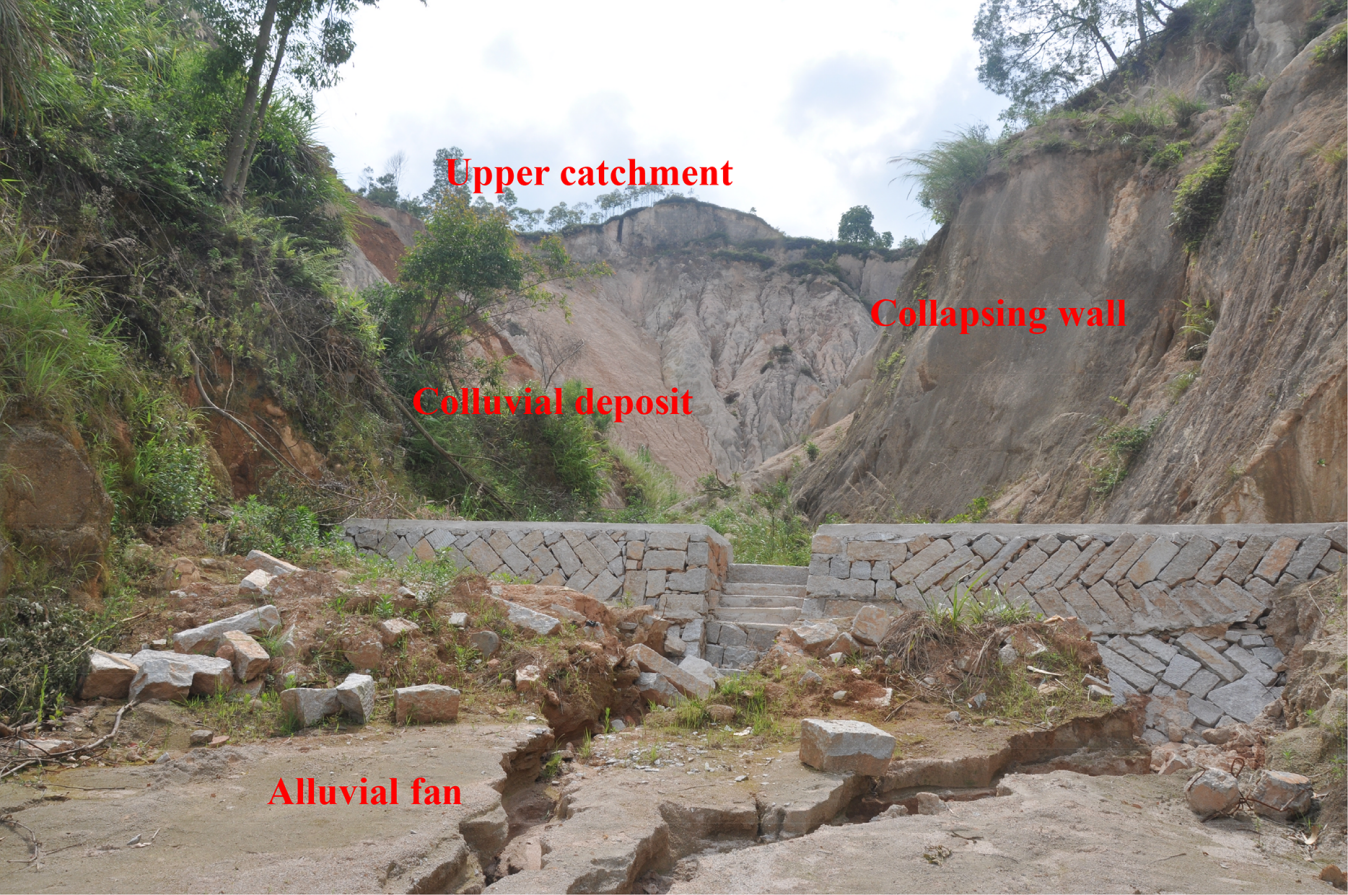
Images of Benggang, also called collapsing gully, in the study area. Benggang has four components: an upper catchment, a collapsing wall, a colluvial deposit, and an alluvial fan. Photo from Fangshi Jiang.

A typical collapsing gully can be divided into four components: upper catchment, collapsing wall, colluvial deposit and alluvial fan (Fig. 1) (Jiang et al., 2018). Collapsing walls are very prone to collapse, mainly because the deep weathering profile sometimes exceeds 20 m, the weathering layer contains high particle content, and the probability of erosion is relatively high (Luk, DiCenzo & Liu, 1997; Jiang et al., 2014). Therefore, the collapse of collapsing walls is the most critical process in Benggang development creating extensive headward erosion and exacerbating soil loss. The stability of the collapsing wall is directly affected by the quantity of the colluvial material and the area of the collapsed trench and determines the severity of the erosion of Benggang (Deng et al., 2018). A similar erosional landform called Lavaka occurs in Madagascar (Cox et al., 2010; Wells, Andriamihaja & Rakotovololona, 1991). Lavaka has dozens of meters of thick weathering granite crust, high contents of coarse particles, poor soil structure, and high water permeability, collapse easily (Cox et al., 2010; Brosens et al., 2022). Therefore, exploring the mechanism of collapse of collapsing walls is important for managing the erosion of collapsing gullies.

Soil is a highly complex, unsaturated medium, and the water in soil systems is a vector for material transfer (Grayson & Western, 1998; Corradini, 2014). The soil water content is a key factor in many causes of wall collapse. With increasing soil water content, soil cohesion decreases significantly, and the internal friction angles between soil materials also decrease (ÇokÇa & Tilgen, 2010), which significantly affects the soil shear strength (Xu, 1996) and the instability of the soil mass of a collapsing gully. In the study of soil water movement, especially in quantitative research, SWCC is a very key index. To date, many scholars have studied SWCCs. The SWRC can either be measured in the laboratory or predicted using a grain-size distribution curve taking into account dry density, porosity, void ratio, and so on (Gupta & Larson, 1979; Aubertin et al., 2003; Zhang, Mavroulidou & Gunn, 2017). Wen et al. (2020b) showed that the water retention capacity of silt and clay decreased with increasing number of drying-wetting cycles. Zhao et al. (2020) measured SWCCs during wetting and drying using a pressure plate instrument in multistep outflow experiments and proposed a simplified equation for the main wetting and drying curves, based on the inkbottle effect and entrapped air effects. Wen et al. (2021) pointed out that the void ratio of soil is affected by its shrinkage behavior, and thus affects the SWCC. The SWCC has also been applied in modeling flow in unsaturated soils and obtaining other unsaturated soil properties, such as shear strength and permeability (Leong, 2019). On the other hand, it can accurately show the relationship between soil water content and matric suction (Oh et al., 2012; Miguel & Bonder, 2012), it is helpful for estimating properties such as shear strength (Neris et al., 2013; Hedayati et al., 2020). The above review shows that investigation of SWCCs is important for analyzing soil water movement and elucidating collapse mechanisms.

The SWCCs of collapsing gullies are closely related to soil properties. Xia, Wei & Cai (2017) obtained the best fit to Benggang SWCCs using the van Genuchten (VG) model, where the model fitting parameters (*a* and *n*) for the SWCCs were positively correlated with sand content. Gu et al. (2021) reported that the Fredlund & Xing model produced the optimal fit for Benggang SWCCs, and the model fitting parameters (*a*, *n* and *θ*_s_) decreased when the soil texture changed from sand to clay. Deng et al. (2018) reported that the suction stress is highly significantly and correlated with the contents of kaolinite, iron oxide and correlated with the contents of hydromica, calcium, and coarse particles. Deng et al. (2018) also showed that the water-holding capacity of residual granite soil decreases with increasing soil depth. Previous studies have mostly used remolded soils with gravels sifted out, so they did not examine gravel in their analysis (Xie et al., 2018; Chai & Khaimook, 2019). However, gravel plays an important role in analyzing the soil water holding capacity. Gravel can significantly affect the soil structure and pore size distribution, thus affecting soil water characteristics (Alberts et al., 1995). Furthermore, the morphology of soil particles affects the pore characteristics (Jiang et al., 2017), which in turn has the opposite effect on SWCCs. However, the effect of particle morphology on SWCCs has seldom been considered in previous studies of collapsing gullies. The analysis presented above shows that the factors affecting the SWCCs of collapsing gullies are complicated. It is necessary to further study the SWCCs of collapsing gullies.

We sampled undisturbed soils in the profiles of two typical collapsing gullies and then used a pressure plate meter to conduct drying experiments on the soil samples. The key goals of the experiments were (1) to fit SWCCs using the VG model and determine the main soil factors that affect the fitted parameters, (2) to analyze the changes in the water-holding capacity of different soil horizons of collapsing walls, and (3) it fully shows the relationship between SWCCs and the collapse of the collapsing walls.

## Materials and Methods

### Study area

The study area is in Yangkeng Village, Longmen Town, Anxi County, Fujian Province (118°03′E, 24°57′N). It has a typical subtropical monsoon climate with a mild climate year round. Precipitation is plentiful, and heavy rainfall is common. The annual average temperature is basically maintained at about 19 °C, and the annual average precipitation is 1,800 mm. The main vegetation in the area is subtropical rainforest. Due to the influence of daily human activities, the community characteristics of primary vegetation are no longer observable. It has been mostly replaced by secondary forests. *Pinus massoniana* is the dominant tree species in the area. The understory plants are mainly *Dicranopteris dichotoma*, *Eriachne pallescens*, and *Syzygium buxifolium*. The soil type in this region is dominated by red soils developed from the parent rock of coarse-grained granite, classified as Ferralsol under the United States Department of Agriculture (USDA) classification, and the soil structure is loose. The rocks weather rapidly under the influence of the subtropical climate, forming a thick regolith largely consisting of quartz, orthoclase, and kaolinite. When there is little vegetation, the thick and loose regoliths are extremely prone to erosion under the effects of precipitation, runoff, and gravity, forming collapsing gullies. A total of 1,228 collapsing gullies are found in Longmen Town, representing 10% of the total number in Anxi County. The collapsing gullies in Longmen are highly eroded and extremely hazardous, with representative characteristics of the Benggang areas in southeast China.

### Sample collection

**Figure 2:**
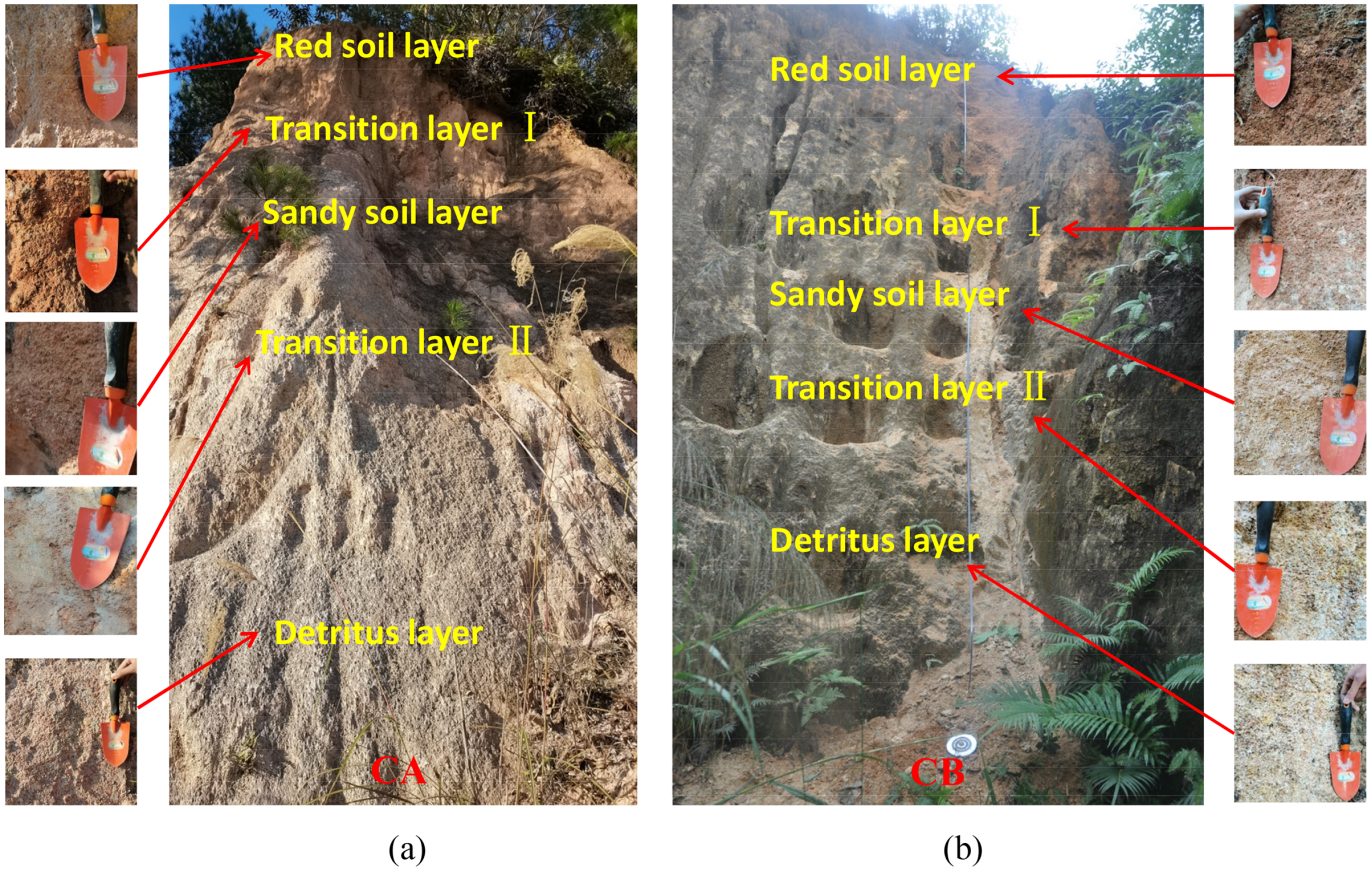
Photograph of soil profile sample collection for collapsing walls CA (A) and CB (B). Photo from Fangshi Jiang.

**Figure 3:**
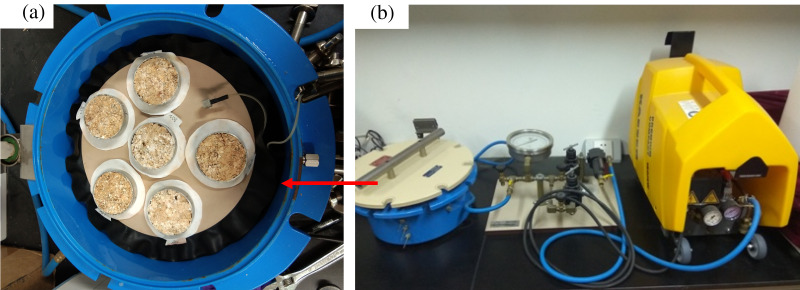
Determination of the soil-water characteristic curve using a pressure plate meter. (A) Soil samples in the test box. (B) The pressure plate meter. Photo from Fangshi Jiang.

We selected two collapsing walls that were well developed in the study area, recorded as collapsing gully CA and collapsing gully CB. The vegetation coverage of CA is 60%, and the height of collapsing wall is 5.1 m. The vegetation coverage of CB is 50%, and the height of collapsing wall is 6.3 m. The soil profile horizons were carefully identified based on the soil color, texture, and vegetation roots. Samples were collected based on the horizons (Fig. 2). Soil samples were collected in May 2020, during the rainy season. A total of 10 samples were collected at collapsing wall CA and eight at collapsing wall CB. The sampling depths are shown in Table 1. After scraping off the first 20 cm of the collapsing gully surfaces, the samples were taken from bottom to top based on the soil horizons. In the depth range of each soil horizon, undisturbed soil samples and mixed soil samples were collected. Cutting rings with a diameter of 61.8 mm and a height of 20 mm were used to collect undisturbed soil samples (Fig. 3A). Repeated sampling was required for each horizon, and three samples were collected for each. A total of 54 soil samples were used for determining SWCCs. Mixed soil samples were obtained by mixing samples collected by multipoint sampling. A total of 18 mixed soil samples were collected, and the detailed physical and chemical properties of all samples were tested and analyzed.

**Table 1:** Chemical properties, elemental and mineral contents of different soil layers.

Soil layers	Number	Soil depth (m)	pH	Organic matter	Element content (g kg^−1^)						Mineral content (%)		
				(g kg^−1^)	Si	Al	Fe	K	Ca		Quartz	Kaolinite	Illite
Red soil layer	CA1	0.10	4.10	9.42	85.10	20.13	12.06	10.43	0.22		71.97	25.48	2.55
	CA2	0.35	3.98	5.28	73.62	23.05	13.24	7.15	0.24		61.54	35.90	2.56
	CA3	0.70	4.54	3.06	73.17	23.74	13.42	9.04	0.35		74.83	22.38	2.80
Transition layer I	CA4	1.00	4.91	2.46	78.02	20.03	12.91	10.08	0.28		72.49	24.89	2.62
Sandy soil layer	CA5	1.35	4.68	1.61	80.75	19.20	13.31	9.38	0.26		79.72	17.48	2.80
	CA6	1.85	4.98	2.16	82.39	18.87	12.60	8.03	0.24		80.75	16.72	2.53
	CA7	2.35	4.82	1.71	84.52	18.91	10.66	12.63	0.32		78.82	17.23	3.95
Transition layer II	CA8	2.80	5.12	0.93	88.58	18.06	8.41	15.62	0.35		64.44	29.65	5.91
Detritus layer	CA9	3.45	4.94	0.91	91.70	17.52	7.79	26.68	0.35		59.79	34.02	6.19
	CA10	4.95	4.76	0.83	100.90	14.60	7.42	28.62	0.44		60.63	33.33	6.04
Red soil layer	CB1	0.20	3.74	3.97	77.93	20.94	12.63	21.44	0.41		70.30	25.74	3.96
	CB2	0.60	3.95	3.78	87.53	28.29	13.20	20.23	0.33		60.02	35.80	4.17
Transition layer I	CB3	1.50	4.56	1.49	81.91	25.24	10.08	23.83	0.35		58.25	37.50	4.25
Sandy soil layer	CB4	2.00	5.38	1.08	112.46	24.68	7.81	29.51	0.34		75.48	20.65	3.87
	CB5	2.50	5.44	0.82	91.32	19.87	8.43	34.38	0.48		72.26	22.06	5.68
Transition layer II	CB6	3.20	5.45	0.78	75.30	19.51	8.22	31.80	0.44		68.60	25.20	6.20
Detritus layer	CB7	4.65	4.86	0.51	109.61	15.41	8.05	41.66	0.55		64.50	29.56	5.94
	CB8	6.10	5.12	0.86	94.48	19.36	7.15	40.47	0.61		63.34	30.79	5.86
Average value in red soil layer			4.06	5.10	79.47	23.23	12.91	13.66	0.31		67.73	29.06	3.21
Average value in transition layer I			4.74	1.98	79.97	22.64	11.50	16.96	0.32		65.37	31.20	3.44
Average value in sandy soil layer			5.06	1.48	90.29	20.31	10.56	18.79	0.33		77.41	18.83	3.77
Average value in transition layer II			5.29	0.86	81.94	18.79	8.32	23.71	0.40		66.52	27.43	6.06
Average value in detritus layer			4.92	0.78	99.17	16.72	7.60	34.36	0.49		62.07	31.93	6.01

### Soil property testing

To measure soil pH, a STARTER 2100 pH meter was used frequently, and a composite glass electrode was used in the measurement process. The soil-water ratio was usually 1:2.5. The redoximetric method with potassium dichromate was mainly used to determine organic matter content. The chemical elements in the soil samples were determined using an X-ray fluorescence spectrometer (XRF). An X-ray diffractometer was used to measure the type and relative content of clay minerals. (KYOWAGLAS-XATM H-12; Kuraray Co., Ltd, Tokyo, Japan). The soil porosity was measured by the cutting-ring method. The soil particle size distribution and morphology for <2-mm fractions were determined using a particle size and shape analyzer (QICPIC, Clausthal-Zellerfeld, Germany). The soil particle size and morphology for ≥2 mm fractions morphology were measured by a WinRHIZO (Pro. 2004c). The specific surface area was measured using a TriStar II 3020 automatic specific surface area and porosity analyzer. The cutting ring method was chosen to measure bulk density. The properties of collapsing wall soil are presented in Tables 1 and 2.

**Table 2:** Physical properties, particle size distribution and morphology of different soil layers.

Soil layers	Number	Physical property				Particle size distribution (%)				Particle morphology		
		Bulk density	Total porosity	Capillary pores	Non-capillary pores	Gravel >2 mm	Sand 2-0.05 mm	Silt 0.05-0.002 mm	Clay <0.002 mm	Aspect ratio	Sphericity	Specific surface area (m^2^ g^−1^)
Red soil layer	CA1	1.38	47.92	28.88	24.21	13.01	40.58	32.61	13.80	0.67	0.77	22.76
	CA2	1.39	47.55	30.15	21.27	11.20	43.94	35.51	9.35	0.69	0.78	26.56
	CA3	1.40	47.17	31.68	16.41	9.05	51.95	31.95	7.15	0.66	0.76	21.46
Transition layer I	CA4	1.37	48.30	36.16	12.58	18.49	48.87	27.60	5.04	0.65	0.75	16.91
Sandy soil layer	CA5	1.36	48.68	34.55	14.85	17.60	48.35	30.44	3.61	0.66	0.76	16.50
	CA6	1.35	49.06	34.44	14.96	17.10	47.20	31.83	3.87	0.64	0.74	18.36
	CA7	1.34	49.43	36.94	12.79	17.90	41.25	36.70	4.14	0.63	0.71	9.98
Transition layer II	CA8	1.55	41.51	22.84	19.63	19.18	45.51	32.24	3.07	0.63	0.70	9.52
Detritus layer	CA9	1.56	41.13	27.64	14.84	23.10	45.29	29.43	2.18	0.62	0.72	8.27
	CA10	1.56	41.13	26.25	15.89	19.63	49.64	28.26	2.47	0.62	0.71	8.01
Red soil layer	CB1	1.38	47.92	29.60	20.49	5.22	56.32	30.23	8.23	0.65	0.74	21.81
	CB2	1.45	45.28	26.35	19.43	18.06	51.83	23.71	6.39	0.66	0.74	19.80
Transition layer I	CB3	1.35	49.06	30.10	20.62	10.65	47.14	33.35	8.85	0.65	0.73	21.22
Sandy soil layer	CB4	1.34	49.43	29.28	20.79	22.96	50.15	22.31	4.58	0.65	0.73	11.53
	CB5	1.38	47.92	36.51	11.57	20.50	36.16	38.76	4.57	0.63	0.72	12.86
Transition layer II	CB6	1.52	42.64	29.29	14.17	20.47	33.90	40.51	5.12	0.62	0.72	10.51
Detritus layer	CB7	1.56	41.13	25.53	16.61	23.47	35.98	36.30	4.24	0.64	0.70	7.92
	CB8	1.57	40.75	25.67	16.15	29.49	33.53	34.10	2.87	0.61	0.70	4.87
Average value in red soil layer		1.40	47.17	29.33	20.36	11.31	48.92	30.80	8.98	0.67	0.76	22.48
Average value in transition layer I		1.36	48.68	33.13	16.60	14.57	48.01	30.48	6.95	0.65	0.74	19.07
Average value in sandy soil layer		1.35	48.90	34.34	14.99	19.21	44.62	32.01	4.15	0.64	0.73	13.85
Average value in transition layer II		1.54	42.08	26.07	16.90	19.83	39.71	36.38	4.10	0.63	0.71	10.02
Average value in detritus layer		1.56	41.04	26.27	15.87	23.92	41.11	32.02	2.94	0.62	0.71	7.27

### Determination and model fitting of SWCCs

The SWCC describes the relationship between water content and water matric suction of a soil. This curve reflects the relationship between soil water potential and quantity. SWCCs was measured using a pressure plate meter manufactured by Soil Moisture Equipment Corp (Fig. 3). All experiments were carried out in the Science and Technology Research Center for Soil and Water Conservation at Fujian Agriculture and Forestry University. The pressure plate meter includes three parts: the pressurization system, pressure chamber, and drainage system. During the testing process, we ensured a tight seal and smooth airflow throughout the entire unit. Before the experiments, the undisturbed soil samples collected by the cutting rings were immersed in water for 24 h to become saturated. Additionally, the ceramic plate was placed in water to absorb water until saturated, and then the surface of the ceramic plate was blotted with filter paper. When experiment started, the ceramic plate was placed in the chamber of the pressure plate meter. Saturated soil samples were placed on the ceramic plate, and put a filter paper between ceramic plate and soil sample, the filter paper can effectively prevent the fine soil particles blocking the ceramic plate, and we ensured that the soil was in full contact with the ceramic plate. By adjusting the pressure valve, 10 suction levels, 5, 10, 20, 40, 80, 150, 300, 600, 1,200, and 1,500 kPa, were applied. When no water had drained from the outlet for 24 h, the sample was considered to have reached equilibrium. The sample was weighed at each suction level. After the samples reached equilibrium at the highest suction level, the final soil samples were transferred to an oven for baking at 105 °C and dried until constant weight. All the samples were weighed, and the water content of samples in the highest suction state was finally calculated. The soil water contents under other suction levels were calculated, and the SWCC was plotted. The indoor temperature was maintained at 25 °C, and each set of experiments was repeated in triplicate.

There are many models for fitting SWCCs. The VG model is a physical and empirical model. Although this model has many parameters and a complicated form, it has a high fitting accuracy. Therefore, it is widely applied in fitting SWCCs. The fitting formula for the VG model is (Van Genuchten, 1980):

(1) $$\theta = ({\theta _s} - {\theta _r})/{[1 + {(a\psi)^n}]^m} + {\theta _r}$$

where *θ*, *θ*_*s*_ and *θ*_*r*_ represent the volumetric water content, saturated water content and residual water content, respectively. $\psi$ is the matric suction in kPa, and the main fitting parameters are *a*, *m*, and *n. m* is usually defined as 1-1/*n* (*n* > 1). Table 3 shown the fitted parameters of the VG models of different soil layers of collapsing walls.

**Table 3:** Fitting parameters of the Van Genuchten model and the statistical parameters used to evaluate the model performance.

Soil layers	Number	*a* (1/kPa)	*n*	*θ*_s_	*θ*_r_	*R* ^*2*^	*NSE*	*p*	*RE* (%)	*MRE* (%)	*MARE* (%)
Red soil layer	CA1	0.045	1.216	0.337	0.188	0.992	0.959	<0.01	−10.8 to 3.9	−6.7	6.7
	CA2	0.046	1.145	0.383	0.174	0.990	0.987	<0.01	−3.0 to 1.1	−0.3	0.7
	CA3	0.035	1.255	0.356	0.197	0.986	0.981	<0.01	−4.3 to 1.5	−0.4	0.9
Transition layer I	CA4	0.026	1.458	0.359	0.182	0.993	0.991	<0.01	−4.0 to 1.8	−0.4	1.0
Sandy soil layer	CA5	0.023	1.624	0.273	0.109	0.971	0.940	<0.01	−12.6 to 3.4	−2.1	3.3
	CA6	0.019	1.549	0.299	0.126	0.956	0.920	<0.01	−14.6 to 3.9	−2.3	3.5
	CA7	0.018	1.590	0.272	0.105	0.956	0.915	<0.01	−15.3 to 4.4	−2.6	3.9
Transition layer II	CA8	0.034	1.382	0.349	0.079	0.994	0.988	<0.01	−6.2 to 3.5	−1.2	2.0
Detritus layer	CA9	0.054	1.329	0.309	0.083	0.986	0.977	<0.01	−9.0 to 1.9	−1.0	1.9
	CA10	0.066	1.275	0.315	0.060	0.989	0.981	<0.01	−8.5 to 1.8	−1.0	1.6
Red soil layer	CB1	0.048	1.406	0.376	0.159	0.968	0.923	<0.01	−10.0 to 1.4	−1.7	2.2
	CB2	0.056	1.268	0.394	0.252	0.962	0.910	<0.01	−9.2 to 1.1	−1.5	1.9
Transition layer I	CB3	0.053	1.273	0.310	0.204	0.971	0.922	<0.01	−6.9 to 0.9	−1.2	1.5
Sandy soil layer	CB4	0.048	1.368	0.286	0.202	0.957	0.915	<0.01	−8.1 to 1.0	−1.4	1.7
	CB5	0.014	1.683	0.371	0.138	0.945	0.900	<0.01	−17.4 to 5.4	−3.2	4.7
	CB6	0.009	1.516	0.386	0.129	0.958	0.931	<0.01	−12.6 to 4.9	−2.2	3.7
Transition layer II	CB7	0.010	1.434	0.363	0.067	0.957	0.929	<0.01	−14.4 to 3.9	−2.5	3.8
Detritus layer	CB8	0.016	1.604	0.378	0.037	0.956	0.918	<0.01	−20.9 to 8.7	−3.5	6.0
Average value in red soil layer		0.046	1.258	0.369	0.194						
Average value in transition layer I		0.040	1.366	0.335	0.193						
Average value in sandy soil layer		0.024	1.563	0.300	0.136						
Average value in transition layer II		0.022	1.449	0.367	0.104						
Average value in detritus layer		0.036	1.411	0.341	0.062						

The following statistical parameters were used to evaluate the performance of the VG model, and that are defined below: the relative error (*RE*, %), mean relative error (*MRE*, %), mean absolute relative error (*MARE*, %), coefficient of determination (*R*^2^), and Nash-Sutcliffe model efficiency (*NSE*). The formulas for calculating these parameters are given in Nash & Sutcliffe (1970) and Jiang et al. (2018).

### Calculation of pore indicators and water-holding capacity indicators

#### Calculation of pore indicators

Soil pores can be envisaged as cylindrical capillaries of various diameters. For simplicity, the soil matric suction *s* and capillary diameter *d* can be expressed as (Lei, Yang & Xie, 1988):

(2) $$s = {4\delta/d}$$

where *δ* represents the coefficient of surface tension of water and is generally kept at 7.5 × 10^−4^ N cm^−1^ at room temperature. When the matric suction *s* is in Pa and the pore diameter *d* is mm, the relationship between them is *d* = 300 *s*^−1^. The calculated pore diameter is the equivalent pore diameter. Suppose that the soil matric suction is *s*_*1*_, the soil water content is *θ*_1_, the equivalent pore size is *d*_1_. and there is also a corresponding relationship between *s*_2_, *θ*_2_ and *d*_2_. According to the analysis, the ratios of the pores should be between *θ*_1_ and *θ*_2_.

To facilitate the analysis of the pore distribution patterns of collapsing gullies, this study divided soil matric suctions into low matric suction (0–100 kPa), medium matric suction (100–500 kPa), and high matric suction (500–1,500 kPa), and their corresponding equivalent pore sizes were >3.00 μm, 0.60–3.00 μm, and 0.20–0.60 μm, respectively (Deng et al., 2016). The equivalent pore distribution ratios of different soil layers from the collapsing walls are shown in Table 4.

**Table 4:** Equivalent pore distribution ratios of different soil layers (%).

Soil layers	Number	Equivalent pore/μm		
		>3.00	0.60–3.00	0.20–0.60
Red soil layer	CA1	11.60	3.68	1.78
	CA2	8.21	3.44	1.66
	CA3	8.57	3.80	1.68
Transition layer I	CA4	11.74	5.42	1.92
Sandy soil layer	CA5	19.32	5.37	1.74
	CA6	21.08	5.90	1.98
	CA7	19.93	5.95	1.96
Transition layer II	CA8	21.39	7.06	3.08
Detritus layer	CA9	18.18	5.04	2.40
	CA10	20.08	5.17	2.65
Red soil layer	CB1	15.20	2.85	0.98
	CB2	25.27	3.19	1.12
Transition layer I	CB3	10.74	1.93	0.76
Sandy soil layer	CB4	11.90	1.87	0.70
	CB5	21.48	9.37	2.67
	CB6	14.07	9.40	4.56
Transition layer II	CB7	16.20	9.71	4.75
Detritus layer	CB8	29.47	12.61	3.93
Average value in red soil layer		13.77	3.39	1.44
Average value in transition layer I		11.24	3.68	1.34
Average value in sandy soil layer		18.74	5.69	1.81
Average value in transition layer II		17.73	8.23	3.82
Average value in detritus layer		20.98	8.13	3.43

#### Calculation of water-holding capacity indicators

According to the range of soil matric suction, the soil water content can be divided into three categories: gravitational water (<30 kPa), available water (30–1,500 kPa), and unavailable water (>1,500 kPa). The soil water content at a matric suction of 30 kPa is usually considered the field capacity, and that at a matric suction of 1,500 kPa is considered the wilting point (Deng et al., 2016). Gravitational water is the difference between saturation and field capacity. Here, available water is the difference between field capacity and wilting point. Unavailable water is the soil water content below the wilting point (Lei, Yang & Xie, 1988). The soil moisture contents of different soil layers from the collapsing walls are shown in Table 5.

**Table 5:** Soil water content of different soil layers.

Soil layers	Number	Gravity water (<30 kPa) (%)	Effective water (30–1,500 kPa) (%)	Non-available water (>1,500 kPa) (%)
Red soil layer	CA1	9.08	7.99	26.03
	CA2	6.16	7.15	28.80
	CA3	6.15	7.90	25.42
Transition layer I	CA4	7.59	11.50	21.59
Sandy soil layer	CA5	14.39	12.04	12.69
	CA6	16.67	12.29	15.57
	CA7	15.51	12.33	12.99
Transition layer II	CA8	15.32	16.21	13.93
Detritus layer	CA9	13.47	12.15	13.53
	CA10	15.38	12.53	13.07
Red soil layer	CB1	12.34	6.68	17.99
	CB2	22.09	7.48	28.23
Transition layer I	CB3	8.85	4.58	23.23
Sandy soil layer	CB4	10.02	4.46	21.93
	CB5	15.49	18.02	17.23
	CB6	10.31	17.72	19.79
Transition layer II	CB7	12.03	18.63	16.23
Detritus layer	CB8	20.86	25.16	9.35
Average value in red soil layer		11.16	7.44	25.29
Average value in transition layer I		8.22	8.04	22.41
Average value in sandy soil layer		14.42	11.83	16.08
Average value in transition layer II		12.82	16.96	16.86
Average value in detritus layer		15.44	17.12	13.04

### Statistics

Data processing and calculation of descriptive statistics were conducted in Excel 2010. The SWCCs were plotted and fitted using Origin 9.0. The analysis of correlations between different parameters and stepwise regression were performed in SPSS 18.0.

## Results

### Soil properties

The pH of the different soil layers ranged from 3.74 to 5.45, and the organic matter content ranged from 0.51 to 9.42 g kg^−1^. With increasing depth, the pH increased overall, whereas the organic matter content decreased (Table 1). Elemental analysis indicated the highest content for silicon, followed by aluminum, iron, and kalium, and the lowest content for calcium. With increasing depth, the contents of silicon, kalium, and calcium increased overall, whereas the contents of iron and aluminum declined (Table 1). The mineral identification results showed the highest content for quartz, followed by kaolinite, and the lowest content for illite. With increasing depth, the content of quartz increased first and then decreased, but the content of kaolinite decreased first and then increased, the sandy soil layer showed the highest content of quartz and lowest content of Kaolinite, and the illite content increased overall (Table 1).

**Figure 4:**
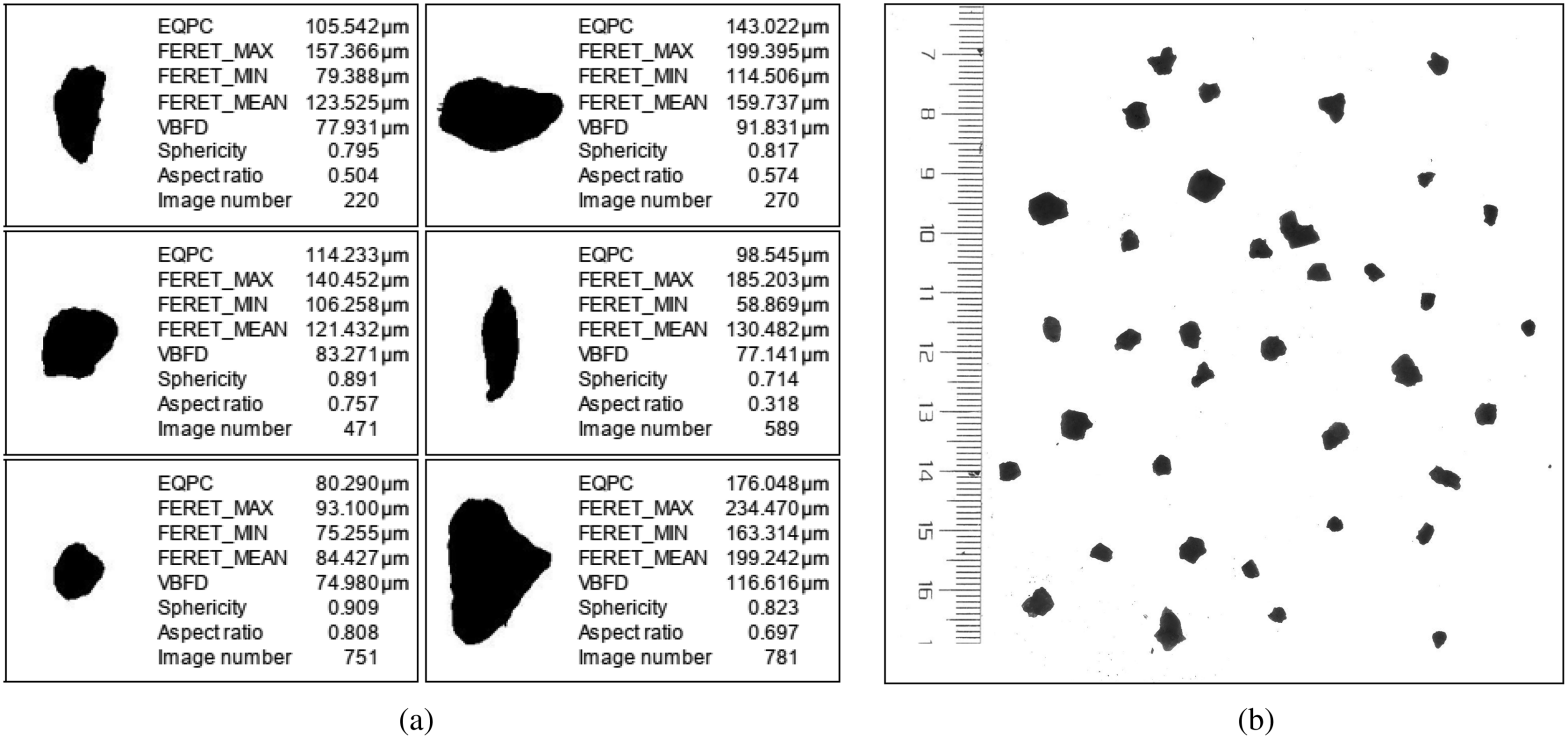
Soil particle characteristics. (A) Morphology of soil particles in <2-mm fractions by QICPIC. (B) Morphology of soil particles in ≥2-mm fractions by WinRHIZO.

The monotonic change in the particle size composition revealed an overall increase, and the gravel content increased obviously, while the clay content tended to decrease (Table 2). The particle aspect ratio ranged between 0.61 and 0.69, indicating that the particles were not spherical, but angular (Fig. 4). The particle sphericity varied between 0.70 and 0.80, corresponding to angular particles (Fig. 4). The specific surface area of the soil particles ranged from 5.07 to 23.91 m^2^ g^−1^. All three morphological parameters declined with increasing depth (Table 2).

Results showed that the soil bulk density was 1.31–1.58 g cm^−3^, and the total porosity was 41.81–50.72%. The bulk density of the lower soil (transition layer II and the detritus layer) was higher than that of the upper soil (the red soil layer, transition layer II and the sandy soil layer), whereas the porosity exhibited the opposite trend. The capillary porosity ranged from 22.84% to 36.94%, and the non-capillary porosity ranged from 11.57% to 24.21% (Table 2). There were no discernible differences between the capillary pore sizes of different soil layers, but the non-capillary pore sizes decreased with increasing depth. The proportion of pore sizes >3.00 μm ranged from 8.21% to 29.47%, the proportion of pore sizes of 0.60–3.00 μm ranged from 1.87% to 12.61%, and proportion of pore sizes of 0.20–0.60 μm ranged from 0.70% to 4.75% (Table 4). Proportion of pore sizes >3.00 μm and 0.60–3.00 μm increased with greater depth, whereas the proportion of pore sizes of 0.20–0.60 μm exhibited the opposite trend.

### Fitting SWCCs

**Figure 5:**
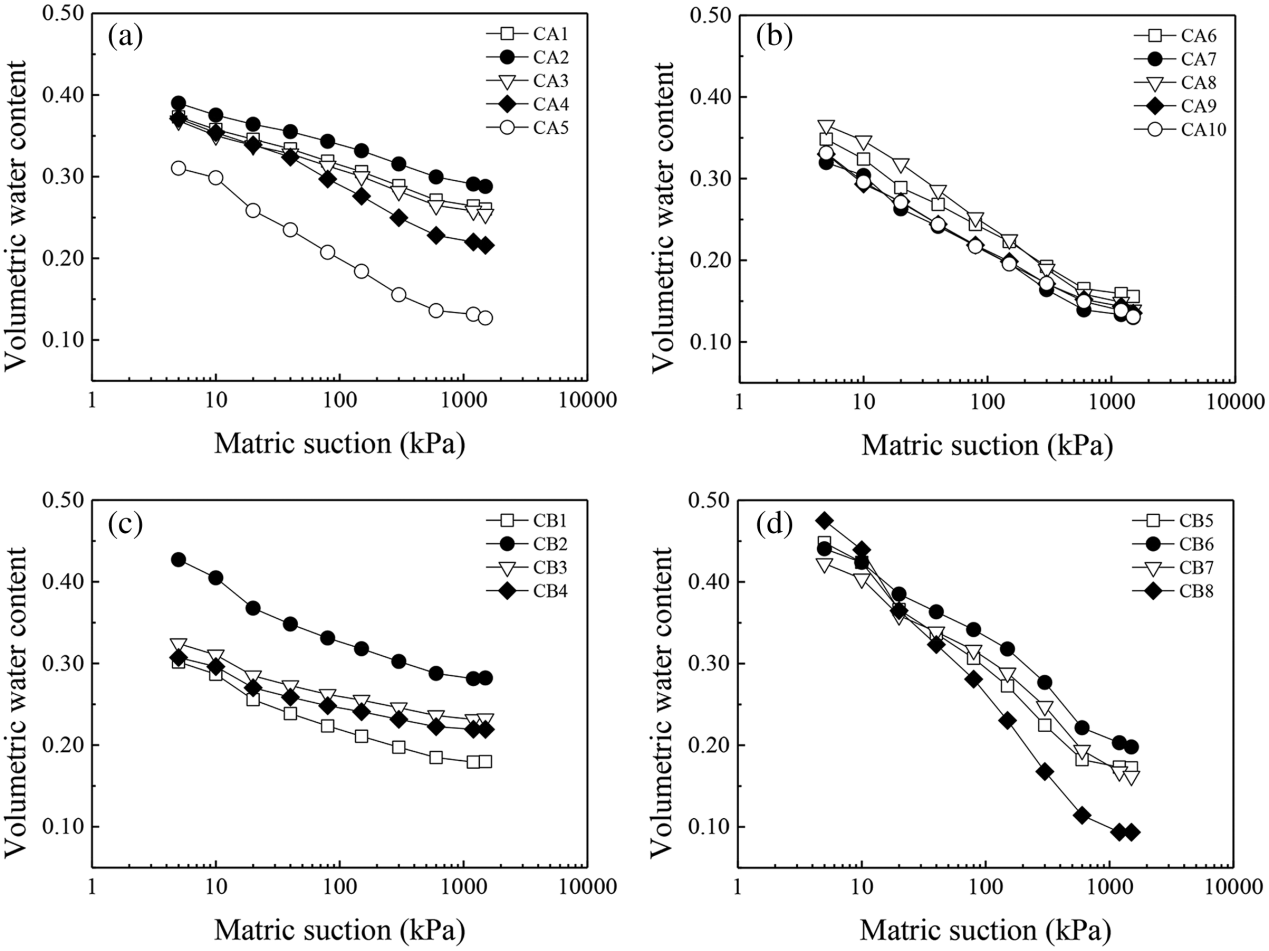
Measured soil-water characteristic curves for different soil layers in collapsing walls. (A) the measured soil-water characteristic curve of CA1, CA2, CA3, CA4, and CA5 in collapsing wall CA; (B) the measured soil-water characteristic curve of CA6, CA7, CA8, CA9, and CA10 in collapsing wall CA; (C) the measured soil-water characteristic curve of CB1, CB2, CB3, and CB4 in collapsing wall CB; (D) the measured soil-water characteristic curve of CB5, CB6, CB7, and CB8 in collapsing wall CB.

Figure 5 shows the SWCCs of different soil horizons. The curves obtained in this study were similar to typical SWCCs (Fredlund, Xing & Huang, 1994; Lu & Khorshidi, 2015). The SWCC can be classified into three successive regimes: a boundary-effect stage, a transformation stage, and a residual stage (Malaya & Sreedeep, 2012). Figure 5 indicates clear variations among the different SWCCs. During the boundary effect stage, water content did not change significantly with increasing matric suction. The inflection points for the red soil layer, transition layer I and sandy soil layer reflecting a transition to the transformation stage all appeared at approximately 10 kPa, whereas the inflection points for the other soil layers appeared later, between 20 and 40 kPa. During the transformation stage, as the matric suction increased and the water content decreased rapidly, the rate of decrease in water content varied among the soil layers, and the slope of the curve increased with increasing soil depth. The water content declined slowly with increasing matric suction, and inflection points for transition layer II and the detritus layer appeared at approximately 600 kPa, reflecting entry into the residual stage. In comparison, the water contents of other layers decreased rapidly over a short period of time with increasing matric suction and remained in the transformation stage.

**Figure 6:**
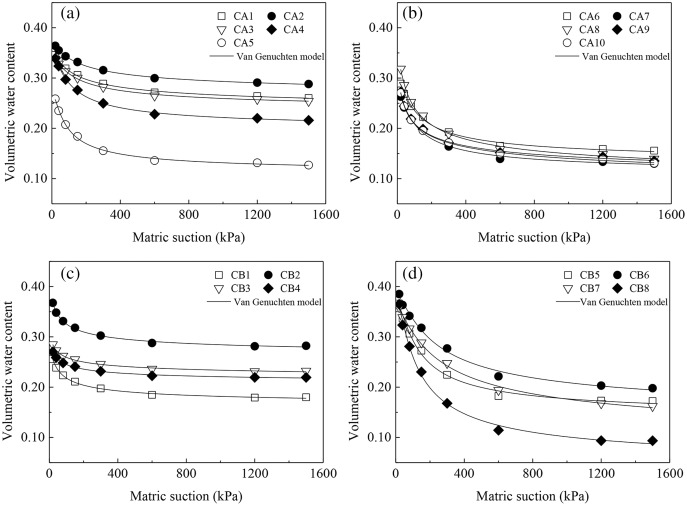
Fitted soil-water characteristic curves for different soil layers in collapsing walls obtained using the van Genuchten model. (A) The fitting curve of CA1, CA2, CA3, CA4, and CA5 in collapsing wall CA; (B) the fitting curve of CA6, CA7, CA8, CA9, and CA10 in collapsing wall CA; (C) the fitting curve of CB1, CB2, CB3, and CB4 in collapsing wall CB; (D) the fitting curve of CB5, CB6, CB7, and CB8 in collapsing wall CB.

The SWCCs were fitted with the VG model (Fig. 6), and Table 3 shows the relevant information for the fitted parameters. The *R*^2^ and *NSE* values of the fitted equation for each soil horizon all exceeded 0.92 and 0.90 (>0.80), respectively, and the *p* values were smaller than 0.01, reaching the extremely significant level. Table 4 shows that the *MRE* ranged from −6.7% to −0.3% and the *MARE* ranged from 0.7–6.0%. These results indicate a good fit by the VG model that meets the study objectives. Xia, Wei & Cai (2017) also reported good fits by the VG model to all the curves for granite soils with different degrees of weathering in Hubei Province in China, whereas Gu et al. (2021) found that the Fredlund & Xing model produced the optimal fit.

The fitting parameter *a* is the reciprocal of the air-entry suction. The larger *a* is, the smaller the air entry value is. The size of parameter *n* is affected by the pores in the soil and changes with its distribution. The more evenly distributed the pore sizes are, the steeper the curve. The saturated water content *θ*_s_ and residual water content *θ*_r_ are also two important parameters, that are closely related to the soil water holding capacity. The larger *θ*_s_ and *θ*_r_ are, the higher the water-holding capacity is (Kong, Sayem & Tian, 2017). Table 3 shows that *a* varied widely between 0.009 and 0.104. With increasing soil depth, *a* decreased first and then increased. The parameter *n* varied between 1.145 and 1.683. With increasing soil depth, *n* first increased and then declined. *θ*_s_ ranged between 0.299 and 0.433. With increasing soil depth, *θ*_s_ generally decreased first and then increased. *θ*_r_ varied widely between 0.037 and 0.252 and declined noticeably with increasing soil depth.

### Water-holding capacities of different soil layers

Figure 5 shows the measured soil-water characteristic curves for different soil layers in collapsing walls, where some regularity in the variations can be observed. Soil water content is closely related to water retention capacity, when change in soil water content is slowest, its water retention capacity is the strongest, resulting in rapid water transport. Figure 5 shows the sandy soil and detrital sediment in the middle and lower horizons contained significantly less water than did the red soil in the upper horizon under the same matric suction. Moreover, Table 3 indicates that the average *a* of the two collapsing walls increased in the order transition layer II < sandy soil layer < detrital layer < transition layer I < red soil layer, whereas the average *n* decreased in the order sandy soil layer > transition layer II > detrital layer > transition layer I > red soil layer. These results indicate that the water releasing capacity was highest for the sandy soil layer and transition layer II, followed by the detrital layer and transition layer I, and the red soil layer had the lowest water releasing capacity. Table 3 also shows that the average θs decreased in the order red soil layer > transition layer II > detrital layer > transition layer I > sandy soil layer.

In addition, we classified the soil water contents, as shown in Table 5. Overall, there was a high gravitational water content in the two collapsing gullies, as high as 22.09%. This result indicated that the overall permeability of the two collapsing gullies was high. The effective contents of the sandy soil layers were relatively high in the two failing gully sections and reached approximately three times those of the red soil layers. The unavailable water contents of red soils were relatively high, almost twice those of sandy and clastic soils. In conclusion, these results showed that soil water-holding capacity decreased with increasing soil depth. After precipitation, the red soil layer was able to store more water than the sandy soil and detrital layers.

## Discussion

### Responses of the soil properties to the degree of weathering

A subtropical monsoon climate generates a high degree of weathering of granite, and a deep weathering crust of up to 50 m forms (Xu, 1996). However, the degree of weathering varies with depth, which produces a well-defined pattern of change elemental composition, particle distribution, and each layer of soil in the profile shows different physical and chemical properties. The results of this study show that as the soil depth increases, the contents of iron and aluminum decrease, whereas the contents of illite, silicon, kalium, and calcium exhibit the opposite trend. As the depth of the soil increases, the clay particle content decreases and the coarse particle content increases. These phenomena mainly occur because as the soil depth increases, there is a decrease in the degree of weathering, as well as the influence of external organisms, temperature, and other factors, and the easily differentiated minerals in the granite cannot be decomposed. Thus, the organic matter and clay particle contents all decline as the soil depth increases. The soil in the surface layer is completely weathered, and clear desilication and aluminization result in a decrease in the silicon content, but the contents of iron and aluminum increase. This result is basically the same as thos of Xu (1996) and Chen et al. (2018). In addition, there were significant differences in the soil properties of different layers. As the soil depth increased, the degree of soil weathering declined, the fine particle content gradually decreased, and the coarse particle content gradually increased (Rahardjo et al., 2004). The contents of organic matter, iron oxide, and alumina in sandy and clastic layers were obviously less than those in the red soil layers. The study results also revealed that the degree of weathering is reflected in the particle morphology, where the overall sphericity, aspect ratio and specific surface area decreased with increasing soil depth, which has not been considered in previous studies. Therefore, the morphology and arrangement of the particles often determine the soil properties of a collapsing wall, and the relationship between the particle morphology and the development of granite weathering requires further study.

### Effects of the soil properties on the fitted parameters of the soil-water characteristic curve

The VG model fits in this study indicated that the *a* parameter was significantly or extremely significantly positively correlated with kaolin content and sand content (*r* = 0.563, *p* < 0.05; *r* = 0.666, *p* < 0.01), negatively correlated with quartz and silt contents (*r* = −0.492, *p* < 0.05; *r* = −0.648, *p* < 0.01) (Table 6). The *n* parameter was significantly or extremely significantly positively correlated with quartz content, capillary porosity, and gravel content and negatively correlated with the contents of organic matter, kaolin, and clay, as well as the non-capillary porosity and specific surface area (Table 6). *θ*_r_ was significantly or extremely significantly positively correlated with the contents of organic matter, iron, aluminum, sand, and clay, as well as the aspect ratio, sphericity, and specific surface area and negatively correlated with illite content, bulk density, and gravel content (Table 6). The correlations between *θ*_s_ and the other parameters were not significant (Table 6). These results are somewhat similar to those found by Xia, Wei & Cai (2017) and many other studies. For example, *a* was negatively correlated with organic matter content and bulk density, and *θ*_r_ was positively correlated with organic matter and clay contents (Niu et al., 2017). The reason is mainly that parameter *a* is affected by the value of the air inlet. Soil texture affects pore sizes and thus changes the air entry (Yang et al., 2004). Parameter *n* was correlated with the evenness of the distribution of pore sizes, which was closely related to soil bulk density and organic matter content. Additionally, increasing contents of organic matter and clay in the soil increased in *θ*_r_.

**Table 6:** Correlation analysis between VG model fitting parameters, soil water content parameters and soil properties.

Parameter	OM	Si	Al	Fe	K	Ca	Quartz	Kaolinite	Illite	BD	TP	CP	NCP	Gravel	Sand	Silt	Clay	AR	SP	SSA
a	0.320	0.076	0.337	0.103	−0.175	−0.322	−0.492*	0.563*	−0.096	−0.046	0.046	−0.429	0.578*	−0.370	0.666**	−0.648**	0.287	0.300	0.260	0.330
n	−0.529*	0.079	−0.364	−0.225	0.272	0.362	0.527*	−0.627**	0.217	−0.017	0.017	0.583*	−0.705**	0.471*	−0.427	0.356	−0.557*	−0.543*	−0.423	−0.498*
*θ*_s_	0.196	−0.226	0.199	0.051	0.261	0.389	−0.446	0.444	0.214	0.386	−0.386	−0.377	0.073	−0.072	−0.221	0.239	0.256	0.081	0.039	0.165
*θ*_r_	0.524*	−0.344	0.876**	0.614**	−0.420	−0.542*	0.084	0.041	−0.610**	−0.660**	0.660**	0.142	0.499*	−0.572*	0.486*	−0.375	0.657**	0.710**	0.661**	0.770**
GW	−0.315	0.321	−0.124	−0.258	0.329	0.373	−0.104	0.025	0.411	0.385	−0.385	−0.087	−0.286	0.519*	−0.138	−0.132	−0.517*	−0.514*	−0.533*	−0.475*
EW	−0.488*	0.228	−0.619**	−0.555*	0.547*	0.698**	−0.060	−0.070	0.623**	0.664**	−0.664**	−0.043	−0.625**	0.712**	−0.759**	0.528*	−0.568*	−0.705**	−0.640**	−0.728**
NAW	0.637**	−0.338	0.746**	0.545*	−0.371	−0.473*	−0.135	0.263	−0.525*	−0.429	0.429	−0.114	0.580*	−0.557*	0.290	−0.185	0.764**	0.787**	0.695**	0.780**

**Notes:**

GW, gravity water; EW, effective water; NAW, non-available water; OM, organic matter; BD, bulk density; TP, total porosity; CP, capillary porosity; NCP, non-capillary porosity; AR, aspect ratio; SP, sphericity; SSA, specific surface area.

* (significant correlation, *p* < 0.05).

** (extremely significant correlation, *p* < 0.01); *n* = 18; If the value is positive, it is positively correlated; otherwise, it is negatively correlated.

Our results also differ from those of previous studies. Xia, Wei & Cai (2017) found a significant negative correlation between water-holding capacity and sand content through a large number of studies. Many investigations have shown that *θ*_r_ is not affected by sand content but is negatively correlated with gravel content. This may be because the presence of gravel weakens the influence of sand grains. In addition, increasing gravel content resulted in higher numbers of large pores in the soil and therefore lower values of *θ*_r_. The results show that the aspect ratio, sphericity and specific surface area increased with the increasing *θ*_r_. The relationships between the fitted SWCC parameters and particle morphological characteristics have not been considered in previous studies.

In summary, this study found that gravel in undisturbed soil exerted a considerable influence on SWCCs. This may be because gravel was able to affect the pore-size distribution of the soil. To date, most studies on the SWCCs of collapsing gullies have used remolded soil from which gravel was sifted during sample preparation, with little consideration given to the influence of gravel (Xie et al., 2018; Chai & Khaimook, 2019). However, gravel is a nonnegligible factor in the study of soil water-holding capacity (Ma & Shao, 2010). Pan et al. (2015) observed that as the gravel content increased, the percolation and drainage of soil water also intensified. Childs & Flint (1990) showed that large number of studies have found gravel to play a positive role in the regulation of soil structure and affect soil porosity and thus water movement. In summary, gravel is important for the study of soil water characteristics. Gravel content is high in the soil of collapsing gullies. Table 2 indicates soil gravel content as high as 30%. The particle morphology can also affect the SWCC. The effect of the aspect ratio and sphericity on the SWCC is due to the increase in the proportion of small pores due to the compactness of the particle contact surface. When the proportion of macropores decreases, the *a* and *n* parameters of the SWCC decrease, and *θ*_r_ increases. There is a close relationship between the specific surface area of soil particles and the adsorption capacity of particles, the larger the specific surface area is, the stronger the adsorption capacity, and the stronger the adsorption of soil water. Thus, soil water is not rapidly discharged, the SWCC flattens, *a* and *n* decrease, and *θ*_r_ increases. Table 2 shows considerable variation in particle morphology among the different soil layers. The particle aspect ratio and specific surface area of the soil particles decrease overall with increasing depth. Therefore, the influence of the gravel content and particle morphology on the SWCCs of collapsing gullies requires further study.

### Effects of the soil properties on the water-holding capacity of profiles of collapsing walls

The study results demonstrated that the water-holding capacity decreased with increasing soil depth. The red soil stored more water, and the sandy soil and detrital layers stored less water. This difference may be attributed to the water-holding capacity being mainly influenced by factors such as the soil particle size composition, soil constitution, and pore sizes (Agus, Leong & Rahardjo, 2001; Zhou, Sheng & Carter, 2012). According to the data in Table 6, gravity water content was significantly positively correlated with gravel content, and significantly negatively correlated with clay content, aspect ratio and so on. Available water content was significantly or extremely significantly positively correlated with gravel content and soil bulk density and negatively correlated with total porosity, non-capillary porosity, clay content, aspect ratio, *etc*. Unavailable water content was significantly or extremely significantly positively correlated with contents of elemental iron, elemental aluminum, capillary pores, and clay, as well as the aspect ratio, sphericity, and specific surface area, and negatively correlated with the non-capillary porosity and gravel content.

The particle size composition and morphology affect the soil water-holding capacity. Under the condition of a certain volumetric water content, the matric suction increases as the soil grains gradually become finer. This pattern occurs because fine soil particles have large specific surface areas and small internal pore spaces, so they have high adsorption and capillary forces, resulting in greater water-holding capacity (Fredlund, 2000; Aubertin et al., 2003). The larger the aspect ratio and sphericity are, the closer the contact surfaces between the particles are, the larger the proportion of small pores in the soil is, and the better the water-holding performance of the soil. The larger the specific surface areas of the particles are, the stronger the adsorption capacity of particles, adsorption effect on the soil water, and water-holding capacity are. The data in Table 2 show that when the depth of soil layer increases, the degree of weathering decreases significantly, the gravel content in the soil increases, and the soil aspect ratio and other parameters decreases, thus the water holding capacity of the soil declines.

The soil constituents, such as organic matter, iron, and aluminum oxide, also affect the water-holding capacity. Declines in the contents of organic matter and aluminum oxide can lead to a decrease in the number of water-stable soil aggregates and deteriorate the soil structure, affecting the water-holding capacity (Nyamangara, Gotosa & Mpofu, 2001; Deng et al., 2018). The data in Table 1 indicate that with increasing soil depth, there are decreases in the degree of weathering and the contents of organic matter, iron, and aluminum, thereby decreasing the water-holding capacity. The soil mineral composition also affects the soil moisture characteristics. For example, the high content of the water-repellent mineral quartz in the sandy soil layer results in favorable water-releasing performance of the soil. Therefore, the sandy soil layer exhibits the highest values of *n*.

Third, the soil water-holding capacity decreases as the proportion of large pores increases (Tisdall & Oades, 1982; Wang et al., 2020). The results of this investigation show the same pattern through analysis of the proportions of equivalent pore sizes of different soil horizons (Table 4). Table 4 shows that the proportions of pores with an equivalent sizes above 3.00 μm and 0.60–3.00 μm were smaller in more weathered laterite than in the sandy soil and detrital layers. There were significantly more 0.20–0.60 μm-sized pores in the detrital layer than in the red soil layer and sandy soil layer. Overall, the proportion of large pores increased with the soil depth.

### Relationships between water characteristics and wall collapse

**Figure 7:**
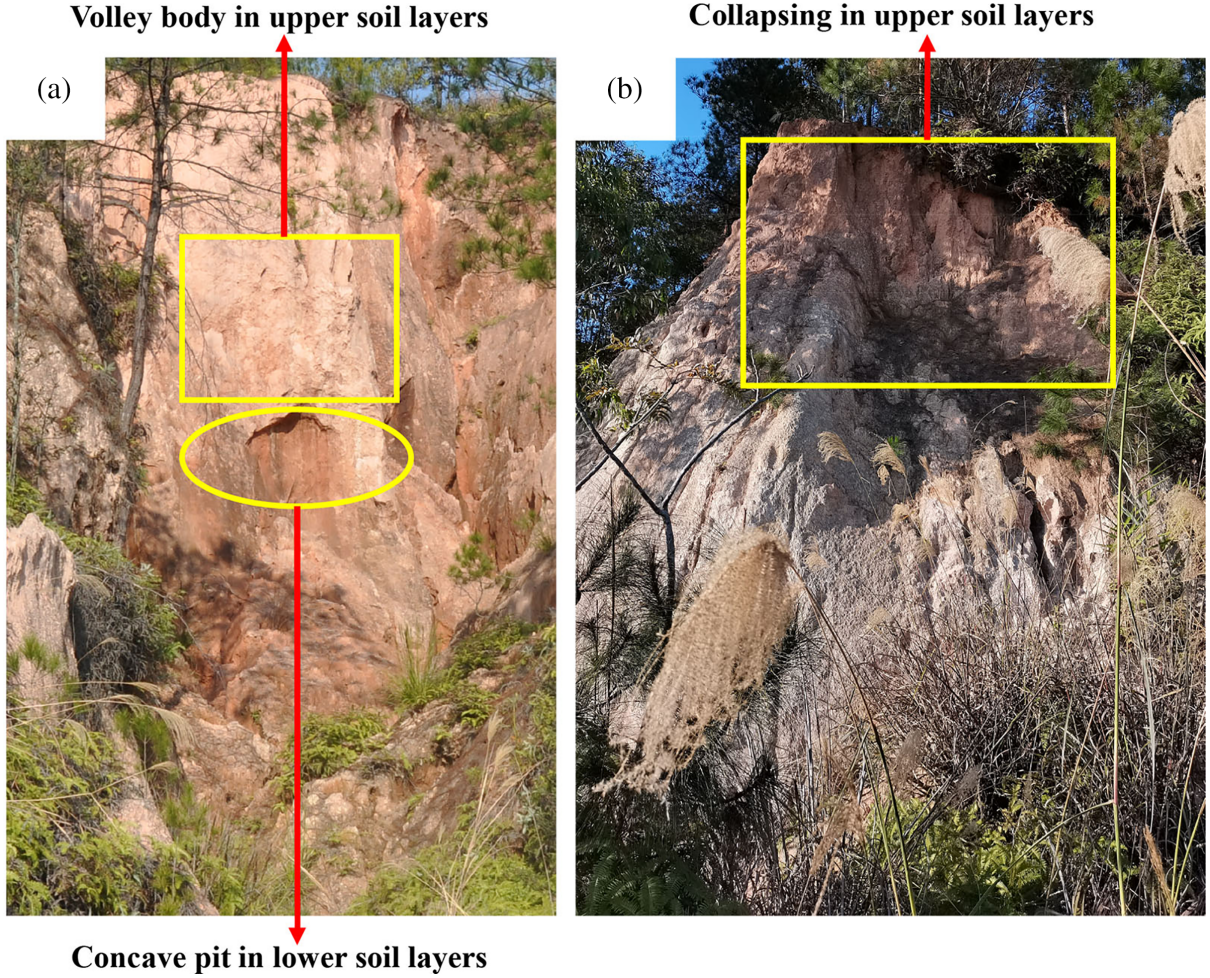
Characteristics of collapsing walls in the field. (A) Concave pit and volley body. (B) Collapse of upper soil layers. Photo from Fangshi Jiang.

The analysis of the water characteristics of the vertical section of the collapsing wall showed considerable variation in the water suction and water-holding characteristics among soil layers, which affects wall collapse. During a concentrated rainfall period, the water content of the soil increases, and the suction of the soil will continues to decrease, which affects the shear strength and stability of the soil to a certain extent, and the overall capacity is significantly reduced (Lin et al., 2015). In particular, the lower portion of the collapsing wall (the sandy soil layer and detrital layer) has poorer water-holding capacity, facilitating rapid saturation of the soil by water. The resulting decrease in the soil shear strength and stability (Xia et al., 2019) allows the wall to collapse easily into a concave pit (Fig. 7A). Thus, the upper layers form a valley (Fig. 7A). In particular, the high suction stress and shear strength of the red soil layer inhibit soil collapse. However, the soil weight increases with the water content. Once the plastic limit and the dead weight of the red soil layer are exceeded (Deng et al., 2016), the entire red soil layer will collapse (Fig. 7B). Deng et al. (2018) also showed that for soil profiles of collapsing walls, the upper layer (the A and B layers) is highly stable, whereas the lower layer (the C layer) is weakly stable and therefore more prone to instability during rainfall, which tends to create a high wall that undergoes deep collapse by gravitation.

The study results also reveal that the red soil layer has the highest degree of weathering and a high content of organic matter and iron oxide. The soil in the top profile has a compact structure and a high water-holding capacity (Xia et al., 2019), which helps to prevent the sandy soil layer and detrital layer below the profile from collapsing (Duan et al., 2021). In Madagascar, Cox et al. (2010) also indicated that the upper solid red clay layer (red soil layer) (0.5–2 m) protects the lower tens-of-meters thick weathered sandy red clay layer (sand layer) and weathered coarse debris layer (debris layer) in the erosional landform of Lavaka. Therefore, human-indeced damage to vegetation and erosion of the red soil layer in granite areas of southern China must be prevented. Erosion of the red soil layer will accelerate the occurrence of Benggang landforms and adversely affect local land use, ecological security, and sustainable development.

## Conclusions

This study takes undisturbed soil as the research object, and the SWCCs can be used to elucidate the mechanism of collapsing gullies collapse. The degree of soil weathering in collapsing walls decreases with increasing soil depth, which is reflected by variation in soil material composition (the amounts of organic matter, elements, and minerals), particle characteristics (the particle size distribution and morphology), and porosity. The SWCCs differed greatly among soil horizons of collapsing gullies, and SWCCs could be accurately described using the VG model. With increasing soil depth, the parameters a and θs first decreased and then increased, and values for the red soil layer were the highest (avg. 0.046 and 0.369, respectively); parameter n tended to increase first and then decrease, with the highest values in the sand layer (avg. 1.563); *θ*_r_ declined as the soil depth increased, and the red soil layer showed the highest values (avg. 0.194). The gravel content of collapsing walls as high as 30%, and gravel content and particle morphology were found to significantly impact the SWCCs. The particle size composition and morphology affect the SWCC, and affect water-holding capacity. With increasing the depth of soil layer, the degree of weathering decreases significantly, the gravel content in the soil increases, and the soil aspect ratio and other parameters decreases, thus the water holding capacity of the soil declines. The lower portion of the collapsing wall has poorer water-holding capacity, the wall to collapse easily into a concave pit, and the upper layers form a valley. Once the plastic limit and the dead weight of the red soil layer are exceeded, the entire red soil layer will collapse. There is still a long way to go to study the soil moisture characteristics of collapsing walls, and future studies should fully consider the impact of the gravel content and particle morphology.

## Supplemental Information

10.7717/peerj.13526/supp-1 Supplemental Information 1 Raw data.

## Additional Information and Declarations

### Data Availability

The following information was supplied regarding data availability: The raw measurements are available in the Supplemental File.
